# Effects of eldecalcitol on cortical bone response to mechanical loading in rats

**DOI:** 10.1186/s12891-015-0613-3

**Published:** 2015-06-30

**Authors:** Yusuke Yamasaki, Keita Nagira, Mari Osaki, Hideki Nagashima, Hiroshi Hagino

**Affiliations:** Graduate School of Medical Sciences, Tottori University, Yonago, Japan; YMCA College of Medical & Human Services in Yonago, Yonago, Japan; Department of Orthopedic Surgery, Faculty of Medicine, Tottori University, Yonago, Japan; Rehabilitation Division of Tottori University Hospital, Yonago, Japan; School of Health Science, Faculty of Medicine, Tottori University, Yonago, Japan

**Keywords:** Eldecalcitol, Mechanical loading, Bone formation, Four-point bending

## Abstract

**Background:**

Mechanical loading of bones activates modeling and suppresses remodeling by promoting bone formation. Eldecalcitol is approved for the treatment of osteoporosis in Japan and is often used in patients undergoing exercise therapy. However, the effects of eldecalcitol on bone formation during mechanical loading are unknown. The aim of this study was to clarify the influence of eldecalcitol administration on bone response to mechanical loading using a four-point bending device.

**Methods:**

Forty six-month-old female Wistar rats were randomized into four groups based on eldecalcitol dose (vehicle administration (VEH), low dose (ED-L), medium dose (ED-M), and high dose (ED-H)). Loads of 38 N were applied *in vivo* to the right tibia for 36 cycles at 2 Hz, by four-point bending, 3 days per week for 3 weeks. After calcein double-labeling, rats were sacrificed and tibial cross sections were prepared from the region with maximal bending at the central diaphysis. Histomorphometry was performed on the entire periosteal and endocortical surface of the tibiae, dividing the periosteum into lateral and medial surfaces.

**Results:**

The effects of external loading on bone formation parameters were significant at all three surfaces. Bone formation parameters were highest in the ED-H group, and the effects of eldecalcitol on bone formation rate were significant at the endocortical surface. In addition, the interaction between loading and eldecalcitol dose significantly affected bone formation rate at the endocortical surface.

**Conclusions:**

Eldecalcitol enhanced the cortical bone response to mechanical loading and a synergistic effect was observed in a rat model.

## Background

Eldecalcitol is a new vitamin D receptor ligand bearing a hydroxypropyloxy substituent at the 2β position of 1α,25-dihydroxyvitamin D3 (calcitriol). While the binding affinity of eldecalcitol to the vitamin D receptor is approximately half that of calcitriol, eldecalcitol binds to serum vitamin D binding protein about four times stronger than calcitriol [1].

In a randomized, placebo-controlled, double-blind clinical trial in osteoporotic patients receiving vitamin D supplementation, 12-month treatment with eldecalcitol increased lumbar and hip bone mineral density in a dose-dependent manner, without causing sustained hypercalcemia [2]. In a fracture prevention trial, 3-year treatment with eldecalcitol reduced the incidence of vertebral fractures to a significantly greater extent than alfacalcidol, with a relative risk reduction of 26 % [3].

Eldecalcitol inhibits *in vitro* osteoclast formation through suppression of c-Fos protein expression in osteoclast precursor cells. Vitamin D also suppresses the expression of nuclear factor of activated T cells c1 (NFATc1), a key regulator of osteoclast formation, by upregulating interferon-β (IFN-β) in osteoclasts [4]. Calcitriol inhibits parathyroid hormone (PTH)-induced bone resorption at physiological concentrations and stimulates bone resorption at toxic doses in thyroparathyroidectomized rats infused with PTH [5]. Based on these findings, eldecalcitol acts mainly as an antiresorptive agent that reduces osteoclast activity. Harada *et al.* recently showed that eldecalcitol inhibits osteoclast maturation and survival by suppressing RANKL expression in osteoblasts on bone surfaces *in vivo*, suggesting that eldecalcitol prevents bone resorption by affecting osteoblast activity *in vivo* [6].

Supplementing these findings, a recent study using an ovariectomized rat model showed that eldecalcitol increased bone formation rate and bone volume, indicating an anabolic effect [7]. Furthermore, histological examination of rat bones treated with eldecalcitol revealed that simultaneous stimulation of differentiation and inhibition of differentiation of osteoblastic cells impaired osteoblast–osteoclast interaction on the bone surface [8].

Mechanical loading of bones activates modeling and suppresses remodeling by promoting bone formation [9]. This process suggests the presence of bone mechanosensors that can transduce mechanical stimuli into anabolic or catabolic signals for bone tissue. Eldecalcitol is approved for the treatment of osteoporosis in Japan and is often used in patients undergoing exercise therapy. However, the effects of eldecalcitol on bone formation during mechanical loading are unknown. The aim of this study was to clarify the influence of eldecalcitol administration on bone response to mechanical loading using a four-point bending device.

## Methods

### Animals

Six-month-old female Wistar rats (retired breeder; Shimizu Laboratory Supply, Kyoto, Japan), with initial body weights ranging between 255 g and 355 g, were used in this study. During the experimental period, water and commercially available food (CE-2; CLEA Japan, Tokyo, Japan; calcium content 1.18 g/100 g, phosphorus content 1.09 g/100 g, vitamin D3 content 250 IU/100 g) were given *ad libitum*. The duration of daily light exposure in the breeding room was 12 h (7:00 AM to 7:00 PM), and the room temperature was maintained at 24 °C.

After a 7-day acclimation period, rats were randomized into four groups based on eldecalcitol dose (n = 10 per group), each with the same mean body weight, as follows: (1) vehicle administration (VEH), (2) low dose (ED-L), (3) middle dose (ED-M), and (4) high dose (ED-H). Rats were allowed normal cage activity between loading sessions.

### Eldecalcitol administration

We prepared 0.025–0.1 μg/ml solutions of eldecalcitol by dissolving in medium-chain triglyceride. Eldecalcitol or vehicle (medium-chain triglyceride) was administered orally via gastric lavage 3 days per week for 3 weeks. The rats received vehicle alone (VEH) or eldecalcitol at doses of 0.025 μg/kg (ED-L), 0.05 μg/kg (ED-M), or 0.1 μg/kg (ED-H). After administration of eldecalcitol or vehicle, tibial mechanical loading was performed on the same day.

### *In vivo* external mechanical loading

*In vivo* mechanical loading involved load application using a four-point bending device (developed and assembled in the Biomechanics Laboratory, Creighton University) [10, 11]. Each rat was anesthetized with ether, and its right lower leg was placed between the pads of the device. The right tibia was loaded at 38 N for 36 cycles at 2 Hz, 3 days per week for 3 weeks, for a total of 9 days. The left tibia was not loaded.

The force applied during loading was monitored by a strain gauge attached to the lever arm as previously reported [10–13]. Before the experiment, the four-point bending device was calibrated with a load cell that had been previously calibrated by application of forces ranging from 0 to 70 N, using a mechanical testing machine (MTS810; MTS, Minneapolis, MN, USA). The actual applied load during *in vivo* four-point bending was calculated based on this calibration [10–13].

### Bone histology

Rats received calcein injections (6 mg/kg BW, i.p.) on experimental days 13 and 19. On day 20, rats in all four groups were anesthetized with 50 mg/kg BW ketamine hydrochloride and 10 mg/kg BW xylazine, and were sacrificed by exsanguination. Both loaded (right) and non-loaded (left) tibiae were removed, placed in 10 % phosphate-buffered formalin for 24 h, and then transferred to 70 % ethanol. The tibiae were cut into three pieces: (a) the proximal 1 cm, (b) the distal 5 mm, and (c) the remaining central diaphysis. Central regions were stained with Villanueva bone stain for 72 h [11, 14]. Specimens were dehydrated with increasing concentrations of ethanol and acetone and then embedded in methyl methacrylate. The region of maximum bending was located in the central diaphysis, 3–13 mm proximal to the tibio-fibular junction (TFJ) [10–13]. Two cross-sections were prepared from the region of maximum bending, specifically at 4 mm and 4.5 mm proximal to the TFJ. These cross-sections were then ground to a thickness of 60 μm and mounted on glass slides. Histomorphometric data were collected from these two sections and mean values were calculated.

### Calculation of *in vivo* strain

The *in vivo* strain was calculated using the moment of inertia of each central diaphyseal cross-section, as previously reported [11]. The outline of the cortical bone on each slide was traced, and the moment of inertia and section modulus for each cross-section were calculated using Bone Histomorphometry Software (System Supply, Nagano, Japan). The peak compressive strain on the lateral surface was calculated using beam-bending theory:

1 $$ \mathrm{\in}\mathrm{c}\kern0.5em =\kern0.5em \mathrm{M}\mathrm{C}/\mathrm{E}\mathrm{I}, $$

where Єc = calculated peak compressive strain on the lateral periosteal surface, M = bending moment (N-m), E = longitudinal Young’s modulus (estimated as 29 × 10^9^ N/m^2^), I = moment of inertia, and C = distance from the centroid to the lateral surface.

The *in vivo* peak compressive strain (Єp) at the lateral periosteal surface was then predicted from Єc using the following formula:

2 $$ \mathrm{\in}\mathrm{p}\kern0.5em =\kern0.5em 0.828*\mathrm{\in}\mathrm{c}\kern0.5em \hbox{-} \kern0.5em 127.16. $$

Equation (2) was derived from the *in vivo* strain gauge measurement [10].

### Histomorphometry

A camera connected to a personal computer was used to run Bone Histomorphometry Software (System Supply Co. Ltd.). The standard nomenclature for bone histomorphometry variables was used [15].

The total tissue area (TtT.Ar, mm^2^) and marrow area (Ma.Ar, mm^2^) were measured, and the difference between them was reported as the cortical area (Ct.Ar, mm^2^). The woven bone contained irregular collagen bundles and a diffuse fluorochrome label, which was identified under conventional polarized and ultraviolet light. The Ct.Ar did not include the woven bone area (Wo.Ar).

For both the periosteum and endosteum, we measured the single-labeled perimeter (sL.Pm, %), double-labeled perimeter (dL.Pm, %), and woven bone perimeter (Wo.Pm, %; defined as the perimeter with overlying woven bone). The Wo.Pm was not included in calculations of the sL.Pm or dL.Pm. The formation perimeter (F.Pm) was defined as (dL.Pm + Wo.Pm + sL.Pm/2)/B.Pm [14, 16, 17]. The mineral apposition rate (MAR; μm/d) and surface-based bone formation rate (BFR; μm^3^/μm^2^/d) were calculated. A minimum MAR value of 0.3 μm/d was used in rats showing only sL.Pm [18]. The BFR was calculated using the formula BFR = MAR × F.Pm [13, 14, 16].

**Fig. 1:**
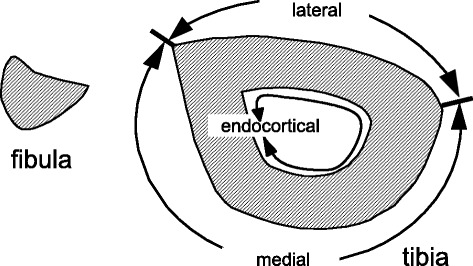
Three surfaces of the tibia for histomorphometric analysis. The tibial periosteal surface was divided into lateral and medial regions as indicated for histomorphometric analysis

Histomorphometric data were collected from the periosteal and endocortical surfaces of the tibia. The tibial periosteal surface was subdivided into lateral and medial surfaces in the same manner as in our previous studies (Fig. 1) [14, 17, 19], because the type of stress applied by the four-point bending device differed between the two surfaces (compression vs. tension) [10].

### Statistical analysis

Data were analyzed by repeated two-way analysis of variance (ANOVA) for the effects of external loading (loaded and non-loaded) and eldecalcitol treatment, and their interaction. Post hoc multiple comparisons among eldecalcitol groups were performed using the Dunnett’s test. SPSS Statistics software (version 21, SPSS Inc., Chicago, IL, USA) was used for the analyses and *P* < 0.05 was considered to be statistically significant.

### Animal ethics

Our procedures were approved by the Committee on Laboratory Animals, Faculty of Medicine, Tottori University, Japan.

## Results

### Body weight

One rat each in the ED-H, ED-L, and VEH groups died during the loading as a result of ether anesthesia. The average initial body weights were 295.7 ± 14.5 g, 293.5 ± 18.6 g, 285.0 ± 8.2 g, and 284.0 ± 15.2 g for the ED-H, ED-M, ED-L, and VEH groups, respectively. The final body weights were 271.5 ± 13.6 g, 275.5 ± 21.0 g, 280.0 ± 18.5 g, and 276.1 ± 10.5 g, respectively. There were no significant differences in initial body weights between the VEH group and the other three groups. There were no significant differences in final body weights between the VEH group and the other three groups, however; there were significant differences between initial and final body weight values in the ED-H (*P* < 0.001) and ED-M (*P* < 0.001) groups.

### Applied force and *in vivo* strain

The monitored mean applied force during loading was 37.5 ± 0.4 N. The applied force, moment of inertia, and *in vivo* peak tibial strain are shown in Table 1. There were no significant differences in these values among the four groups. The variation between strains within each group was due to differences in the tibial moment of inertia in each rat.

**Table 1:** Applied force and *in vivo* peak tibial strain

	VEH	ED-L	ED-M	ED-H
Force (N)	37.5 ± 0.43	37.7 ± 0.4	37.7 ± 0.22	37.2 ± 0.32
Moment of inertia (mm^4^)	1.82 ± 0.22	1.95 ± 0.31	1.81 ± 0.20	1.77 ± 0.25
Compressive peak strain (μstrain)	2240.2 ± 225.8	2127.7 ± 256.9	2182.0 ± 199.2	2283.0 ± 278.1

Compressive peak strain was calculated using beam-bending theory based on each moment of inertia. ED-H: high dose eldecalcitol; ED-M: medium dose eldecalcitol, ED-L; low dose eldecalcitol; VEH: vehicle

### Histomorphometry

**Fig. 2:**
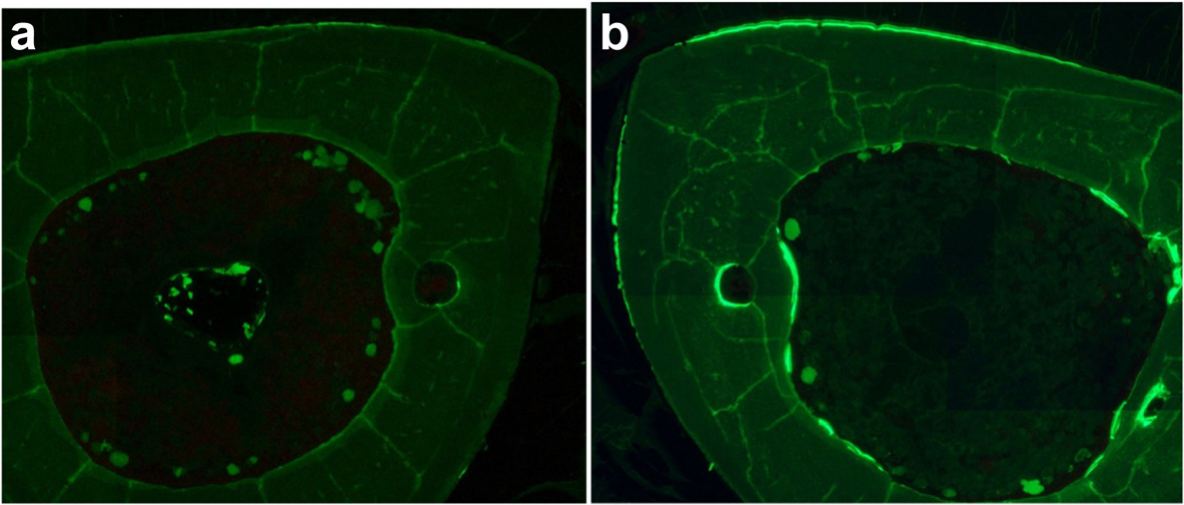
Cross-sections of high-dose eldecalcitol rat. (**a**) Left tibia; (**b**) right tibia. Increased bone formation was observed in the right (loaded) tibia compared with the left (non-loaded) tibia, indicated by increased calcein labeling at both the periosteal and endocortical surfaces

Increased bone formation was observed in cross-sections of loaded tibiae, as greater calcein labeling occurred on both the periosteal and endocortical surfaces than in the non-loaded tibiae (Fig. 2). In three rats in the VEH group, woven bone formation was observed at the lateral and medial periosteal surfaces of the loaded (right) tibiae; however, no woven bone was observed in the ED groups.

#### Cortical area

There were no significant differences in Ct.Ar, TtT.Ar, or Ma.Ar between the loaded and non-loaded tibiae (Table 2). There were no significant differences among the four groups in the loaded and non-loaded tibiae.

**Table 2:** Cortical area measurements

		VEH	ED-L	ED-M	ED-H
TtT.Ar (mm^2^)	loaded	5.6 ± 0.3	5.8 ± 0.3	5.8 ± 0.3	5.5 ± 0.4
	non-loaded	5.6 ± 0.2	5.6 ± 0.2	5.7 ± 0.4	5.5 ± 0.4
Ma.Ar (mm^2^)	loaded	1.8 ± 0.1	1.8 ± 0.1	1.8 ± 0.2	1.7 ± 0.3
	non-loaded	1.8 ± 0.2	1.7 ± 0.2	1.9 ± 0.2	1.7 ± 0.2
Ct.Ar (mm^2^)	loaded	3.9 ± 0.2	4.0 ± 0.2	3.8 ± 0.2	3.8 ± 0.4
	non-loaded	3.8 ± 0.1	4.0 ± 0.3	4.0 ± 0.3	3.7 ± 0.3

ED-H: high dose eldecalcitol; ED-M: medium dose eldecalcitol, ED-L; low dose eldecalcitol; VEH: vehicle

#### Lateral periosteal surface

**Fig. 3:**
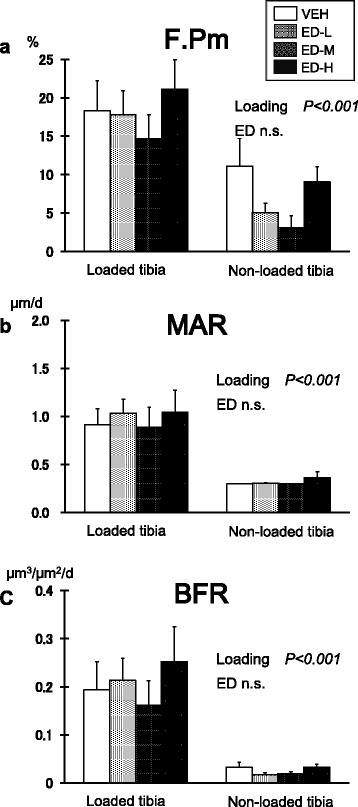
Bone response at the lateral periosteal surface. (**a**) Formation perimeter (F.Pm); (**b**) mineral apposition rate (MAR); (**c**) bone formation rate (BFR). There were significant differences between the loaded and non-loaded sites in all three parameters by repeated two-way analysis of variance (*P* < 0.001). F.Pm and BFR in the high-dose eldecalcitol group were highest among the four groups at the loaded tibiae; however, the differences with the vehicle group were not significant compared by Dunnett’s test. Data are mean ± SEM. ED-H: high dose eldecalcitol; ED-M: medium dose eldecalcitol, ED-L; low dose eldecalcitol; VEH: vehicle

There were significant differences in F.Pm, MAR, and BFR between the loaded and non-loaded sites (*P* < 0.001 by repeated two-way ANOVA) (Fig. 3). F.Pm and BFR were highest in the ED-H group among the four groups at the loaded tibiae; however, the differences were not statistically significant by Dunnett’s test.

#### Medial periosteal surface

**Fig. 4:**
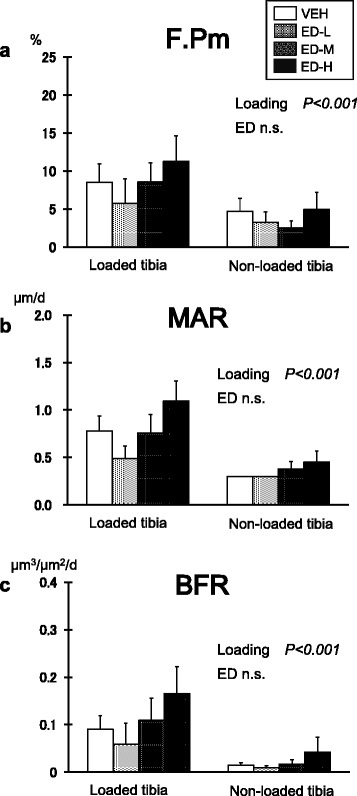
Bone response at the medial periosteal surface. (**a**) Formation perimeter (F.Pm); (**b**) mineral apposition rate (MAR); (**c**) bone formation rate (BFR). There were significant differences between the loaded and non-loaded sites in all three parameters by repeated two-way analysis of variance (*P* < 0.001). F.Pm, MAR, and BFR in the high-dose eldecalcitol (ED-H) group were highest among the four groups at both loaded and non-loaded tibiae; however, the differences with the vehicle group were not significant compared by Dunnett’s test. Data are mean ± SEM. ED-H: high dose eldecalcitol; ED-M: medium dose eldecalcitol, ED-L; low dose eldecalcitol; VEH: vehicle

There were significant differences between the loaded and non-loaded sites in F.Pm, MAR, and BFR (*P* < 0.001 by repeated two-way ANOVA) (Fig. 4). F.Pm, MAR, and BFR were highest in the ED-H group among the four groups at both loaded and non-loaded tibiae; however, the differences were not statistically significant by Dunnett’s test.

#### Endocortical surface

**Fig. 5:**
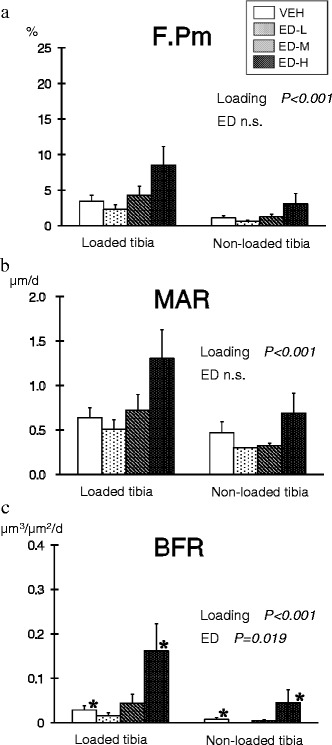
Bone response at the endocortical surface. (**a**) Formation perimeter (F.Pm); (**b**) mineral apposition rate (MAR); (**c**) bone formation rate (BFR). External loading exerted a significant effect on all three parameters (*P* < 0.001 by repeated two-way analysis of variance). F.Pm and MAR in the high-dose eldecalcitol (ED-H) group were highest among the four groups at both loaded and non-loaded tibiae; however, the differences with the vehicle (VEH) group were not significant compared by Dunnett’s test. BFR in the ED-H group was significantly higher than in the VEH group (*P* = 0.019, by Dunnett’s test), and the interaction between loading and the effects of eldecalcitol was significant (*P* = 0.043 by repeated two-way analysis of variance). Data are mean ± SEM. ED-H: high dose eldecalcitol; ED-M: medium dose eldecalcitol, ED-L; low dose eldecalcitol; VEH: vehicle

There was a significant effect of external loading on F.Pm, MAR, and BFR (*P* < 0.001 by repeated two-way ANOVA) (Fig. 5). F.Pm and MAR were highest in the ED-H group among the four groups at both loaded and non-loaded tibiae; however, the differences were not statistically significant. BFR was significantly higher in the ED-H group compared with the VEH group (*P* = 0.019, by Dunnett’s test), and the interaction between loading and eldecalcitol treatment was significant (*P* = 0.043).

## Dis**c**ussion

Mechanical loading accelerates bone formation by stimulating osteoblasts and their precursors via a signal network of osteocytes and osteoblasts. Recent studies on the complex role of osteocytes and sclerostin have begun to shed light on the mechanisms underlying the control of bone mass by loading [20]. Devices for four-point bending of the tibia [11] and for loading of the ulna in the axial direction [21] have been developed to quantitatively measure the degree of strain in bone caused by non-invasive mechanical loading, as well examining the effects of duration and frequency of stress on bone formation. A previous study used a four-point bending device with a loading schedule of 36 cycles at 2 Hz, 3 days per week (the same parameters used in the current experiment), and demonstrated increased bone formation [13]. Consistent with previous work, we observed cortical bone response at both the periosteal and endocortical surfaces [22, 23]. Ours is the first study to evaluate bone response during the simultaneous administration of eldecalcitol and mechanical stimulation. It is also the first to analyze the effects of eldecalcitol at three different doses. In this study, eldecalcitol dose-dependently enhanced bone formation and this enhancement showed interaction with bending effects in BFR at the endocortical surface.

In a preventive study in which eldecalcitol was administered orally once daily at dosages of 0.05 μg/kg, 0.1 μg/kg, and 0.2 μg/kg, dose-dependent improvements occurred in bone mineral density and mechanical properties in an ovariectomized rat model [8]. Based on this model, we used 0.025 μg/kg, 0.05 μg/kg, and 0.1 μg/kg of eldecalcitol 3 days per week. In Japan, eldecalcitol has been approved to treat involutional osteoporosis at a daily dose of 0.75 μg [24]. In this study, the eldecalcitol doses in rats ranged from 0.05 μg/kg to 0.1 μg/kg, both 3 days per week, and were between 3.9- and 15.5-fold higher than the 0.75-μg daily dose administered to a human weighing 50 kg.

We demonstrated that eldecalcitol enhanced bone formation dose-dependently and exerted a synergistic effect on the cortical bone response to mechanical loading at the endocortical surface. Active vitamin D compounds induce receptor-activated of NF-κB ligand (RANKL) expression in osteoblastic cells and enhance osteoclast formation and bone resorption *in vitro* [25]. It has been reported that mice with global VDR knockout as well as those with conditional knockout of VDR in osteoblasts show a higher bone mass with reduced bone resorption; however, bone histomorphometry showed no effect on bone formation parameters [26]. It was reported that eldecalcitol suppressed bone resorption by reducing the number of RANKL-positive cells on the trabecular bone surface [6]. The decrease in bone formation following low-dose eldecalcitol administration is thought to be the result of a coupling reaction induced by the suppression of bone resorption; however, this reaction may be offset by the positive effects of high-dose eldecalcitol on bone formation in response to mechanical loading.

Prior research showed that eldecalcitol administration for 12 weeks increased cancellous bone volume and bone formation rate without affecting bone resorption in aged rats [7]. We previously demonstrated in a rabbit model that distraction osteogenesis with eldecalcitol increased callus volume during the early period after the completion of lengthening, resulting in thick cortical bone formation [27]. In a rabbit model examining expansion of the mid-palatal suture, eldecalcitol had positive effects on bone formation parameters in the early phase of bone regeneration [28]. One research group reported that eldecalcitol reduced osteoclast numbers and diminished osteoclastic activity and function, without promoting osteoclast apoptosis in ovariectomized rats [29, 30]. This group also demonstrated “bud-like” or “bouton-like” bone formation patterns characteristic of bone minimodeling in eldecalcitol-treated ovariectomized rats at rates 10-fold higher than in those treated with calcitriol, and suggested that eldecalcitol stimulates osteoblastic activity at the bone surface *in vivo*. Bone formation in response to mechanical loading is primarily due to modeling of cortical bone. Increased bone formation in the current study suggests that bone modeling of cortical bone could be increased by eldecalcitol treatment. These data demonstrate that eldecalcitol was capable of increasing bone mass not only by suppressing bone resorption, but also by stimulating bone formation. Eldecalcitol increased bone formation at the periosteal surface of SAM/P6 mice, and it is speculated that eldecalcitol activates Wnt signaling and/or growth factor signaling via enhanced muscle function [31]. In the current study it is possible that eldecalcitol suppressed sclerostin expression and activated Wnt signaling caused by mechanical loading. However, the effect of eldecalcitol on sclerostin is still unclear. Two clinical observations reported conflicting results regarding sclerostin changes after treatment with vitamin D [32, 33], and therefore further studies are required to clarify this phenomenon.

We observed a significant increase in bone formation at the endocortical surface. Since the direct effects of the loading pads affect the response at the periosteal surface and woven bone influenced periosteal surfaces, the values of F.Pm were not increased in a dose-dependent manner, which is consistent with previous studies [22, 23]. Compared to the periosteal surface, the preferential endocortical bone response to loading and to eldecalcitol treatment may be due to the lack of a direct pad effect and to the lower induced mechanical strain (stimulus).

There are several limitations to this study. First, the experiments were performed on rats rather than humans. Unlike humans, rat cortical bone has no Haversian system, so cortical bone remodeling is absent. Second, the rats in the model we used were estrogen-replete, and the effects at estrogen-deplete status have not been defined.

## Conclusions

This study used a rat model to assess the interactions between eldecalcitol administration and mechanical loading of cortical bone. Eldecalcitol enhanced the cortical bone response to mechanical loading through a synergistic effect.

## Abbreviations

BFR: Bone formation rate
TtT.Ar: The total tissue area
Ct.Ar: mm2, Cortical area
dL.Pm: Double-labeled perimeter
F.Pm: Formation perimeter
Ma.Ar: mm2, Marrow area
MAR: Mineral apposition rate
sL.Pm: Single-labeled perimeter
Wo.Ar: Woven bone area
Wo.Pm: Woven bone perimeter
